# Role of crystal orientation in attosecond photoinjection dynamics of germanium

**DOI:** 10.1063/4.0000253

**Published:** 2024-08-19

**Authors:** Nicola Di Palo, Lyudmyla Adamska, Simone Bonetti, Giacomo Inzani, Matteo Talarico, Marta Arias Velasco, Gian Luca Dolso, Rocío Borrego-Varillas, Mauro Nisoli, Stefano Pittalis, Carlo Andrea Rozzi, Matteo Lucchini

**Affiliations:** 1 Department of Physics, Politecnico di Milano, Piazza Leonardo da Vinci, 20133 Milano, Italy; 2 CNR – Istituto Nanoscienze, via Campi 213/A, I-41125 Modena, Italy; 3 Department of Physics, Informatics and Mathematics, University of Modena and Reggio Emilia, via Campi 213/A, I-41125 Modena, Italy; 4 Institute for Photonics and Nanotechnologies, IFN-CNR, 20133 Milano, Italy

## Abstract

Understanding photoinjection in semiconductors—a fundamental physical process—represents the first step toward devising new opto-electronic devices, capable of operating on unprecedented time scales. Fostered by the development of few-femtosecond, intense infrared pulses, and attosecond spectroscopy techniques, ultrafast charge injection in solids has been the subject of intense theoretical and experimental investigation. Recent results have shown that while under certain conditions photoinjection can be ascribed to a single, well-defined phenomenon, in a realistic multi-band semiconductor like Ge, several competing mechanisms determine the sub-cycle interaction of an intense light field with the atomic and electronic structure of matter. In this latter case, it is yet unclear how the complex balance between the different physical mechanisms is altered by the chosen interaction geometry, dictated by the relative orientation between the crystal lattice and the laser electric field direction. In this work, we investigate ultrafast photoinjection in a Ge monocrystalline sample with attosecond temporal resolution under two distinct orientations. Our combined theoretical and experimental effort suggests that the physical mechanisms determining carrier excitation in Ge are largely robust against crystal rotation. Nevertheless, the different alignment between the laser field and the crystal unit cell causes non-negligible changes in the momentum distribution of the excited carriers and their injection yield. Further experiments are needed to clarify whether the crystal orientation can be used to tune the photoinjection of carriers in a semiconductor at these extreme time scales.

## I. INTRODUCTION

Ultrashort flashes of light offer nowadays the enticing possibility of manipulating the electronic and optical properties of a solid at extreme temporal scales.^1,2^ As a result, light can be used to encode and process information into a crystal at unprecedented speed (i.e., at frequencies approaching the petahertz)^3–7^ or to study the injection of charge carriers in organic semiconductors for the field of energy harvesting.^8^ However, all these fascinating opportunities rely on a deep understanding of the ultrafast electron dynamics triggered by the interaction between an intense laser pulse and a solid-state system.^9^ The enormous advances in laser technology of recent years have made the generation of few-femtosecond or attosecond pulses a unique tool to study nonlinear light–matter interaction at time scales comparable with the motion of electrons inside matter.^10^ Attosecond transient absorption or reflection spectroscopy^11^ has thus been exploited in the past decade to ultimately observe ultrafast phenomena in solids on their natural time scales, such as the injection of carriers from valence (VB) to conduction (CB) band in a semiconductor,^12–17^ the sub-cycle modification of the optical properties of an insulator,^18–21^ or the creation of a core-excitonic state.^22–24^

For the case of a semiconductor, the photoinjection process initiated by a few-optical-cycle, intense electric field [usually in the near-infrared/visible (NIR/VIS) region of the optical spectrum] constitutes a rather complicated phenomenon with no simple description. In fact, the promotion of carriers from VB to CB, which mainly dictates the optical response of the material, is the result of the complex interplay between different mechanisms taking place during nonlinear light–matter interaction,^9,25^ such as single- or multi-photon absorption, tunneling excitation, band-dressing, or intra-band motion. The role of each of these processes in determining the total excited charge strongly depends on the pump pulse parameters (i.e., field amplitude, photon energy, pulse duration) and the electronic band structure of the material. Therefore, recent experiments employing similar optical schemes to investigate different semiconductors led to substantially different outcomes.

In the first experiment of this class, Schultze and co-workers used attosecond transient absorption spectroscopy (ATAS) to investigate electron dynamics in silicon induced by a few-cycle IR (photon energy of 1.55 eV) pump pulse with an attosecond extreme-ultraviolet (XUV) probe pulse at the $L_{2,3}$ absorption edges of Si.^12^ The experiment allowed to resolve the sub-cycle modification of the bandgap energy (in this case, larger than the IR photon energy), explained with the influence of the carriers promoted in the CB via tunneling excitation. With a similar experiment, a few years later, Schlaepfer *et al.* studied the carrier photoinjection process in gallium arsenide at the As $M_{4,5}$ edges with sub-femtosecond resolution.^13^ With a bandgap energy close to the IR photon energy, they found that photoexcitation originates from single-photon absorption, although the number of excited carriers is significantly increased (by almost a factor of three) by IR-induced intra-band motion.

The case of a narrow-gap semiconductor such as germanium, where the IR photon energy exceeds the direct bandgap energy (0.8 eV), led to a different scenario. Inzani and co-workers investigated the photoinjection dynamics in monocrystalline, undoped germanium by combining attosecond transient reflectivity measurements at the Ge $M_{4,5}$ edges with a double theoretical approach.^17,26^ They found that several mechanisms come into play within the pump pulse envelope, each one with a different timing. While single-photon excitation is predominant during the rising edge of the pump pulse, when the IR electric field reaches its peak both tunneling and multi-photon processes become relevant, with the second dominating at later times. Conversely from what observed in GaAs,^13,27^ field-induced intra-band motion is found to reduce carrier photoinjection by driving different families of k points in and out of resonance. The different role of intra-band motion in determining the ultrafast charge dynamics around the energy gap of a solid has strong consequences on the material electro-optical properties, affecting, for example, its capability to be used as active media for high-order harmonic generation.^28^

Since the complex charge injection mechanism observed in Ge is the result of the specific family of k points involved and the local properties of the band structure, it is natural to explore whether the orientation of the crystal lattice with respect to the IR polarization can be used to modify the physical pictures and the charge injection yield. In light–matter interaction, crystal symmetries generally dictate the angular dependence of the optical response of the material, both in the perturbative^29^ and nonperturbative regime.^30,31^ Therefore, by investigating the role of the relative alignment between the crystal structure and the light electric field, interesting phenomena such as orientation-dependent multiphoton ionization^32^ or anisotropic high-harmonic generation^32–34^ in bulk insulators have been observed. Following the approach presented in Ref. 17, in this work, we studied the effect of the relative crystal alignment on ultrafast photoinjection dynamics in Ge by performing attosecond transient reflection spectroscopy (ATRS) measurements at two distinct configurations. Despite the IR pump exciting and driving the carriers along different directions in the crystal structure, we surprisingly observed that the differential reflectivity of the material does not present qualitative changes, but only an overall increment of the amplitude of the transient features. This was investigated through time-dependent density functional theory (TDDFT) calculations, which allowed us to display the distribution of the carrier population in reciprocal space and to compare the build-up of the total excited charge for the two orientations, demonstrating that the carrier injection mechanism is qualitatively robust in terms of both the involved physical processes and their timing. Finally, a quantitative analysis performed on both numerical and experimental data explains the augmented charge injection efficiency that follows crystal rotation by 45° as originating from both single and double-photon excitation around the Γ point. Despite the overall process remains dominated by two-photon transitions at larger values of the crystalline momentum, the qualitative nature of the population variations is dictated by one-photon transitions, which give a stronger transient contribution. Our results not only elucidate the role of crystal orientation in defining the ultrafast charge injection dynamics in Ge, but they also suggest the strength and limitations of ATRS in the XUV range as a probe for the role of crystalline structure on the observed ultrafast dynamics.

## II. MATERIALS AND METHODS

### A. Sample orientation and experimental parameters

**FIG. 1.**
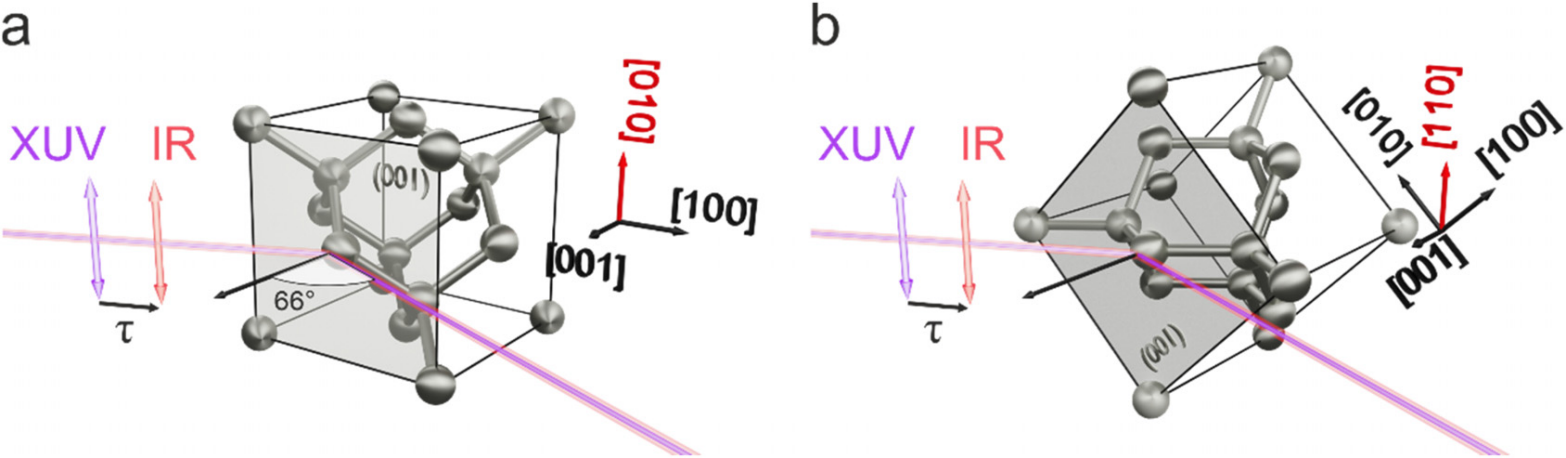
Optical setup and Ge crystal structure. Schematic representation of the Ge crystal cell and the optical setup for the two configurations investigated. In both cases, the free surface of the sample corresponds to the (001) crystal plane. XUV and IR beams are s-polarized and impinge on the sample with an angle of 66° with respect to the surface normal ([001] direction). In the first configuration (a), the field polarizations are aligned along the [010] direction while in the second (b), they are parallel to the [110] direction. τ indicates the delay between IR pump and XUV probe.

The experimental setup employed in this work has been widely described in Ref. 35. Photoinjection of charge carriers from VB to CB of Ge is obtained by pumping the material with ultrashort IR pulses of 5.3±0.8 fs temporal duration (intensity full-width half-maximum, the uncertainty corresponds to the standard deviation over different measurements) centered at 800 nm ( $\hbar \omega_{IR}$ = 1.55 eV). The IR peak intensity is set to 10.0 TW/cm^2^ in vacuum, which translates to 1.2 TW/cm^2^ inside the crystal. The photoinduced changes in carrier population for both VB and CB are then probed with an attosecond pulse train (APT) composed by 2/3 pulses, generated in Krypton via high-order harmonic generation spanning over the 25–45 eV energy range and covering the $M_{4,5}$ absorption edges of Ge, respectively, at 29.2 and 29.8 eV. Both XUV and IR beams are *s*-polarized with respect to the crystal surface and impinge on the crystal with an angle of incidence of 66° with respect to the surface normal, coinciding with the [001] crystal direction (see Fig. 1).

The sample is a commercial, intrinsic Ge wafer (Active Business Company GmbH), where the surface corresponds to the (001) plane and has optical quality. Chemical action by means of a hydrofluoric acid solution ensures the removal of the native oxide layer from the crystal surface. In the first chosen orientation [Fig. 1(a)], the IR and XUV polarization axes are parallel to the [010] direction. We notice that the cubic symmetry of the lattice ensures that the structure is invariant for π/2 rotations around the [001] direction, making the [100] direction equivalent to the one reported in Fig. 1(a). Conversely, a rotation of π/4 around the [001] axis brings the light polarization parallel to the [110] crystal direction [Fig. 1(b)], possibly changing the lattice properties observed by the radiation. For example, the distance between two neighboring atoms along the polarization direction equals the lattice constant a=5.66 Å in the first case, while it reduces to $\frac{a}{\sqrt{2}}=4.00$Å for the second one. In addition, atoms belonging to different planes along the [110] direction are linked in a zigzag chain of bonds. Therefore, the atomic (hence, the electronic) density interacting with the laser electric field substantially increases when going from the [010] to the [110] orientation, possibly affecting the ultrafast charge injection mechanism.

### B. Calculated ultrafast electron excitation

To investigate the effect of the sample orientation on the ultrafast charge injection mechanisms, we computed the time-dependent electron excitation induced by the IR pulse with TDDFT using the Elk software suite^36^ with the PBE+U+J functional.^37–39^ The calculations were performed with the same parameters used in Ref. 17, but considering the shorter IR pulse used in this work and increasing the grid size to 16^3^ points in the Brillouin zone to ensure a proper sampling of the different excitation phenomena. The indirect bandgap of 0.6 eV (in fair agreement with the experimental value of 0.7 eV) is achieved by explicit inclusion of U and J Hubbard parameters on top of a PBE functional as detailed in the SI of Ref. 17. The time-dependent carrier occupation analysis was performed with a step size of 48 as by sampling the projected occupations $p_{ik}\left(t\right)$ for each band i and k-point k using the equation $p_{ik}\left(t\right)=\sum_{j}f_{jk}^{gs}{\left|\left\langle \varphi_{ik}^{gs}|\varphi_{jk}\left(t\right)\right\rangle \right|}^{2}$, where $f_{jk}^{gs}$ are the ground state (GS) occupation numbers, and $\varphi_{ik}^{gs}$ and $\varphi_{jk}\left(t\right)$ are the GS and time-evolved Kohn-Sham orbitals, respectively. The projected occupations take values between 0 and 1. Therefore, to obtain the correct number of total electrons, we calculate the excited state charge per atom as follows: $N_{\text{exc}}\left(t\right)=\frac{1}{N_{k}N_{at}}\sum_{i=CB}\sum_{k=BZ}p_{ik}\left(t\right)$, where $N_{k}$ and $N_{at}$ are the number of k-points and number of Ge atoms per unit cell, respectively.

## III. RESULTS AND DISCUSSION

### A. Theoretical results

**FIG. 2.**
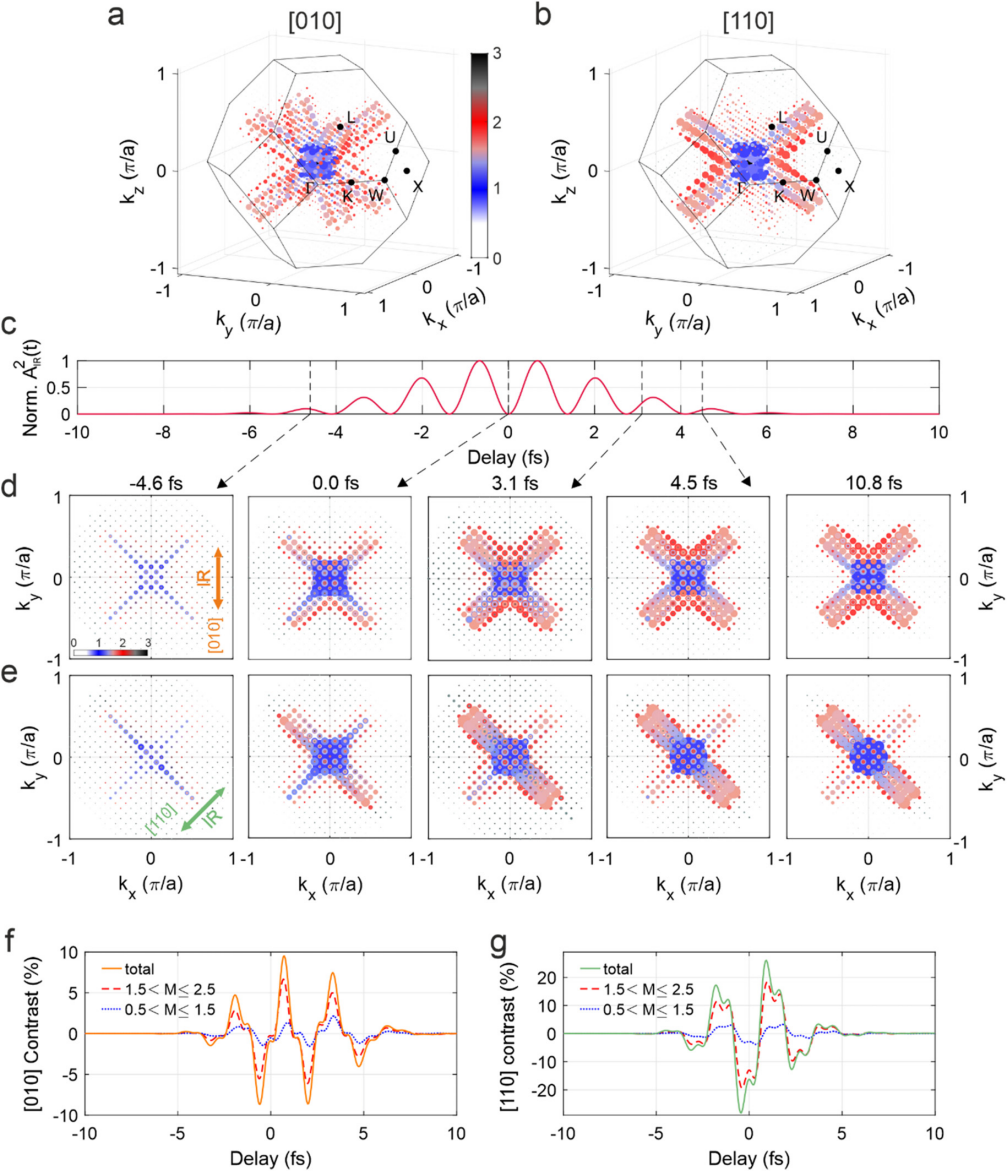
Calculated distribution of the excited k points. (a) Distribution of the k points within the Ge first BZ that present a net population after the interaction with the IR pump pulse for the [010] orientation. The area of the dots is proportional to the net electron excitation while the color scale indicates the width of the local energy gap. The blue scale indicates a gap close to the photon energy (resonant parameter M ∼ 1), while the red and black scales represent k points where the local resonant parameter is M ∼ 2 and 3, respectively. (b) Same as (a), but for the [110] orientation. (c) Squared vector potential of the IR pump field. (d) $k_{x}$- $k_{y}$ projection of the first BZ, taken at different time instants within the interaction with the light pulse for the [010] case. (e) Same as (d), but for the IR field along the [110] direction. In this case, the positive diagonal, ${k_{y}=k}_{x}$, is overall less populated. (f) Relative mirror contrast induced by intra-band motion with respect to the $k_{y}=0$ plane, calculated for the [010] geometry. The full curve is calculated by considering all the active k points. The red dashed curve is based on those points with 1.5<M≤2.5, and the blue dotted curve considers only those points with 0.5<M≤1.5. (g) Same as in (f), but calculated with respect to the $k_{y}=-k_{x}$ plane for the [110] geometry.

The results of the time-dependent electron occupation analysis are summarized in Fig. 2, where panels (2a) and (2b) report the residual excited charge in the first Brillouin zone (BZ), evaluated when the IR pulse is over (i.e., ∼11 fs after the peak of the IR field envelope), for the two different crystal orientations. The size of each dot is proportional to the electron population at each specific k-point, while the color code relates to the energy gap between VB and CB in that specific region of the band structure. Blue dots indicate regions where the resonant parameter M, defined as the ratio between the local energy gap and the IR photon energy,^25^ is close to one (0 .5<M≤1.5). The red (black) dots correspond to points with a gap matching two (three) IR photons, i.e., 1.5<M≤2.5 ( M>2.5). Correspondingly, we dissect the total excited charge by contributions from one-photon and two-photon transitions. This classification is approximate since each k point may simultaneously contribute excited charge from both one-photon transitions and two-photon transitions from deeper valence bands. When a specific k-point is colored in blue (red), it must be understood as the primary contribution to the excited charge at that k-point originates from one-photon (two-photon) transitions. The minimum direct bandgap in Ge—located at Γ—is ∼0.8 eV, so no points with M≤0.5 are present. Moving away from Γ, electrons are excited by single-photon transitions (blue dots), while two-photon absorption dominates for larger values of $\left|k\right|$ (red dots). Excitation due to three-photon processes is barely visible at the edge of BZ in panels (2d) and (2e) (black dots).

As it is possible to observe, the distribution in reciprocal space of the excited electron population changes when the crystal is rotated by π/4 along the [001] direction. While in the [010] case [Fig. 2(a)] the IR almost symmetrically promotes electrons along the eight Γ−L directions in reciprocal space, the excitation is significantly reduced along four of them and enhanced along the others for the [110] orientation, reflecting the π/4 rotation in the $\left(k_{X},k_{y}\right)$ plane. These are the two extreme cases to demonstrate the charge excitation asymmetry. In the case of [110] orientation, the vector field shifts the k-points along the direction of the Ge–Ge bonds, where the charge density (of valence charge) is primarily distributed. In the case of [010] orientation, the direction of vector field points into an empty space, at +45° and −45° to the Ge–Ge bonds, thus exciting the charge along those directions.

This effect becomes clearer when looking at the projection of the populated k points in the $\left(k_{X},k_{y}\right)$ plane, which are reported in Figs. 2(d) and 2(e) for different instants of time during the interaction. The rotation of the light polarization direction not only changes the k points that are effectively populated, but also causes a rotation of the direction along which intra-band motion is observed. In Fig. 2(d), the red points in the panel corresponding to t=3.1 fs are asymmetric with respect to the $k_{y}=0$ plane, perpendicular to the IR field direction. As expected from intra-band motion, this asymmetry is reversed after half cycle of the IR electric field (see the red dots in the panel corresponding to t=4.5 fs). For the [110] orientation, intra-band motion induces instead an asymmetry with respect to the $k_{y}=-k_{x}$ plane [Fig. 2(e)]. Figures 2(f) and 2(g) show the relative mirror contrast for the [010] and [110] orientation, respectively, calculated as the difference of the electron population found on the opposite sides of the asymmetry plane and divided by the total residual population. Due to the different excited k points and the effect of intra-band motion, the temporal evolution of the contrast changes with the orientation. Nevertheless, in both cases, it goes to zero at the end of the interaction with the IR field and it is dominated by those k points that are mostly two-photon resonant (red dashed curves in both panels). A weaker mirror symmetry breaking is observed for the one-photon resonant k points family (blue dotted curves), whose relative amplitude is little affected by the crystal rotation.

**FIG. 3.**
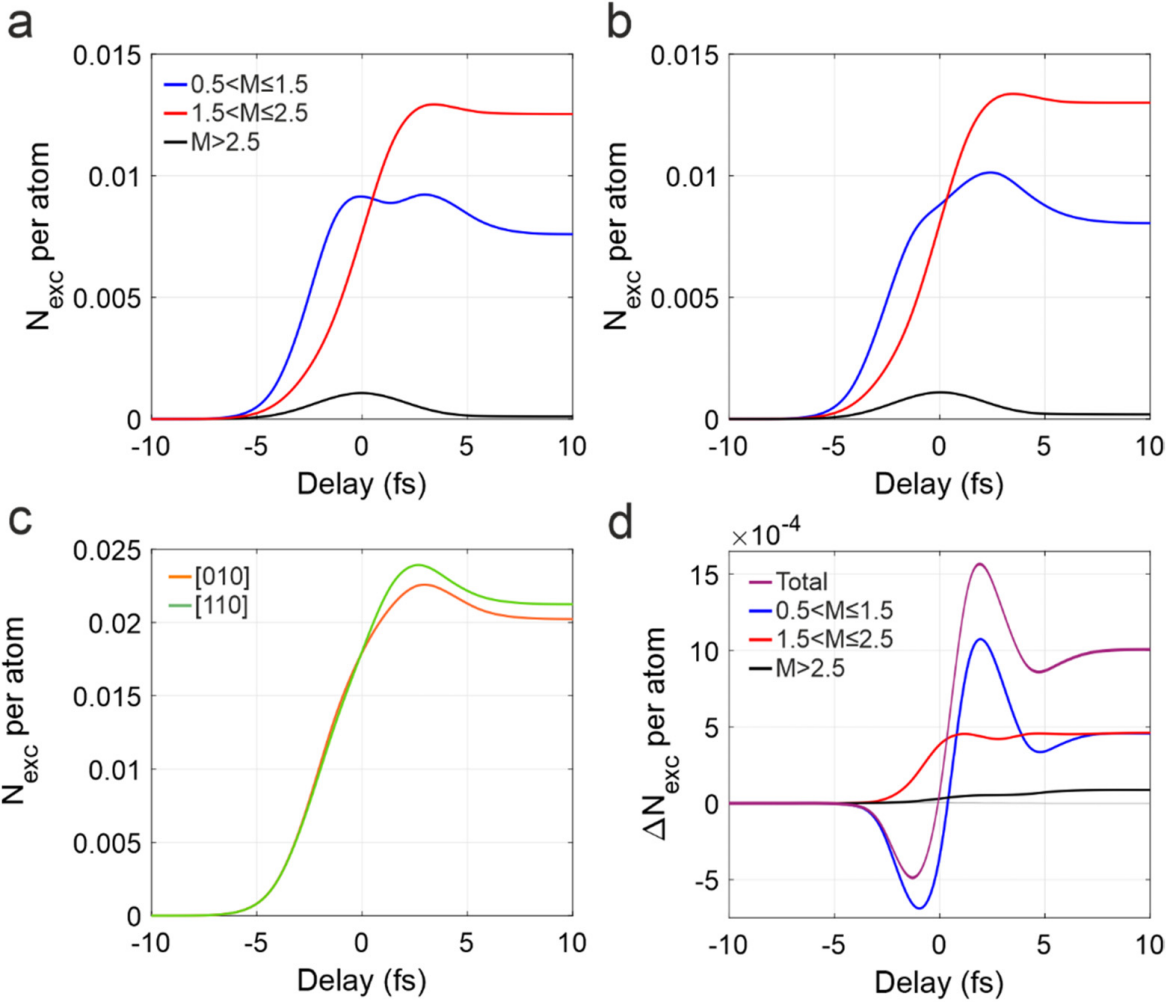
TDDFT temporal evolution of the excited electron population. (a) Calculated temporal evolution of the number of excited electrons per atom, $N_{\text{exc}}$, for the [010] orientation and for the families of k points identified in Fig. 2. A local resonant parameter M between 0.5 and 1.5, between 1.5 and 2.5, and bigger than 2.5 is represented by the blue, red, and black curves, respectively. (b) Same as (a), but for the [110] crystal orientation. (c) Temporal behavior of the total excited population per atom for [010], orange, and [110], green. (d) Difference between the $N_{\text{exc}}$ of the two orientations ([110] minus [010]), considering only a specific family of k points [same color code as in (a) and (b)] and the total population (violet curve). In all panels, the populations are obtained by spline-fitting the TDDFT results corresponding to the instants of time where $A_{\text{IR}}\left(t\right)=0$.

Despite the differences highlighted above, we find the ultrafast charge injection mechanism to be qualitatively the same for both orientations. This is highlighted by the similar temporal evolution of the charge injected into the CB for the different families of k points reported in Figs. 3(a) and 3(b). We note that while the temporal behavior of the transient populations has a qualitative meaning, showing an increasing charge excitation with superimposed 2 ω oscillations, its exact value is gauge invariant only when the IR vector potential, $A_{IR}\left(t\right)$, equals zero. To assure our analysis to be gauge-independent, we hereafter consider only those instants where $A_{IR}\left(t\right)=0$ and perform a spline fit to extract the time evolution of the population reported in Fig. 3. For both the [010] and [110] directions, our results show that single photon excitation around Γ (blue curves) dominates during the leading edge of the pump pulse, while two-photon excitation (red curves) becomes predominant around the peak of the electric field envelope, giving the biggest contribution to the total residual charge injection.^17^ The k points with M>2.5 (black curves) are mostly only transiently populated, giving a non-zero, but negligible contribution to the final population.

As a consequence of the robustness of the photoinjection mechanism, the total injected charge, $N_{\text{exc}}\left(t\right)$, displays a very similar time evolution for the two orientations [Fig. 3(c)]. Nevertheless, the calculations show that [110] orientation is characterized by a higher injection yield, resulting in a 5% larger residual population. Therefore, the theoretical results suggest that crystal rotation can be used both to selectively excite carriers in specific regions of the electronic band structure and to fine-tune the injection efficiency, despite the robustness of the underlying physical mechanism.

To better understand the origin of the yield increment in the [110] geometry we studied the contribution of the different families of k points to the electron population difference between the [110] and the [010] directions, ${\Delta N}_{\text{exc}}\left(t\right)$. The results are reported in Fig. 3(d), where the violet curve indicates the total population, while blue, red, and black refer to the different families of k points identified in Fig. 2, where we presorted the k-resolved excited charges by the primary type of transition. While all population differences are positive at large delays, indicating an overall increased electron excitation, the total population difference (violet curve), starts with negative values and changes its sign around t=0 fs. The same behavior is observed for k points with M∼1 (blue curve), which are found to give a strong contribution to the total ${\Delta N}_{\text{exc}}\left(t\right)$, and originates from the different timing of the one-photon processes, which peak at later time for the [110] case [compare the blue curves in Figs. 3(a) and 3(b)]. Therefore, even though the overall injection mechanism is dominated by two-photon processes [red curves in Figs. 3(a) and 3(b)], the results of Fig. 3(d) allow to identify single-photon injection in the proximity of Γ as the main responsible for the observed derivative shape of the change in population induced upon crystal rotation.

**FIG. 4.**
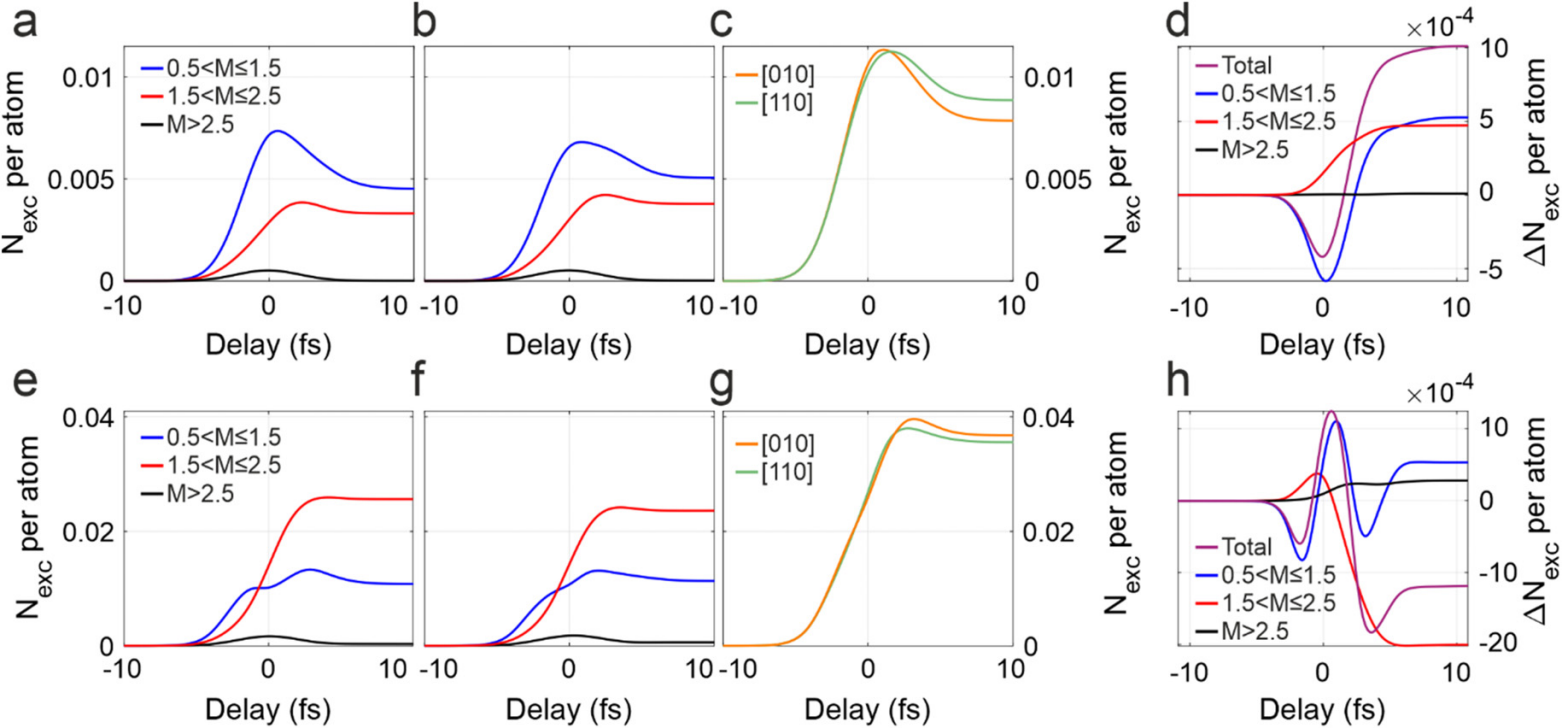
Intensity dependence of the excited electron population. (a) Calculated temporal evolution of the number of excited electrons per atom, $N_{\text{exc}}$, in the [010] geometry and for an IR intensity reduced by 50% (i.e., 5.0 TW/cm^2^). Black, blue, and red curves represent those k families with a local resonant parameter M between 0.5 and 1.5, between 1.5 and 2.5, and bigger than 2.5, respectively. (b) Same as (a), but for the [110] crystal orientation. (c) Temporal behavior of the total excited population per atom for [010], orange, and [110], green. (d) Difference between the $N_{e\text{xc}}$ of the two orientations ([110] minus [010]), considering only a specific family of k points [same color code as in (a) and (b)] and the total population (violet curve). (e)–(h) Same quantities as in (a)–(d) but obtained with an IR intensity increased by 50% (i.e., 15.0 TW/cm^2^). In all panels, the populations are obtained by spline-fitting the TDDFT results corresponding to the instants of time where $A_{\text{IR}}\left(t\right)=0$ as in Fig. 3.

Since the probability for the different multiphoton mechanisms changes with the IR intensity,^17^ and since the relative number of k points that are close to resonance with one or more IR photons varies with the orientation [Fig. 2(a)], the presented physical picture may exhibit a non-trivial dependence on $I_{IR}$. While an experimental investigation of this aspect is hindered by sample damage on the high intensity side and laser source noise on the opposite intensity side,^11^ it can be studied theoretically with TDDFT. Figures 4(a)–4(d) show the same quantities presented in Fig. 3 but calculated with half the IR intensity (5.0 TW/cm^2^). The results reported in Figs. 4(e)–4(h) have instead been obtained by increasing the pump intensity by 50% ( $I_{IR}=15.0$ TW/cm^2^). Lowering the intensity does not qualitatively change the observed dynamics. The contribution of k points with 1.5<M≤2.5 gets stronger after 0 fs [red curves in Figs. 4(a) and 4(b)], which causes the total population after the pulse to be bigger for the [110] case [Fig. 4(c)]. Both the points close to one- and two-photon resonance contribute almost equally to the total ${\Delta N}_{\text{exc}}\left(t\right)$, with the qualitative time behavior that is dictated by the points with 0.5<M≤1.5 [Fig. 4(d)].

At high intensity, two-photon processes clearly dominate [red curves in Figs. 4(e) and 4(f)]. In this case though, the total residual excitation for the [010] orientation [orange curve in Fig. 4(g)] is higher than the one of the [110] case [green curve in Fig. 4(g)]. The total ${\Delta N}_{\text{exc}}\left(t\right)$ becomes negative after the interaction with the pump pulse [violet curve in Fig. 4(h)] and is dominated by the contribution of those k points with 1.5<M≤2.5 [red curve in Fig. 4(h)]. Nevertheless, its qualitative behavior is still dominated by the points close to one-photon resonance [blue curve in Fig. 4(h)], showing a maximum close to time zero and two local minima before and after. The change in sign of ${\Delta N}_{\text{exc}}\left(t\right)$ for $I_{IR}=15.0$ TW/cm^2^ is consistent with the fact that the reciprocal space region that can be excited by the pump is markedly different at the two angles. For the [010] orientation the excited charge is populated along all eight Γ−L directions, which lie on two orthogonal planes. Only half of those, four Γ−L diagonals on one of the orthogonal planes, are populated for the [110] case. As those points are mainly characterized by 1.5<M≤2.5 [red dots in Fig. 2(a)], this results in way more k-points possibly contributing to two-photon absorption [see red dots in Fig. 2(a)] for the [010]. At high IR intensities, the contribution of two-photon processes becomes dominant and, therefore, the total number of excited electrons per atom for the [010] case exceeds $N_{\text{exc}}$ for [110]. These new findings, although purely theoretical, confirm that the efficiency of photo-injection depends in a non-trivial way on the excitation intensity. Moreover, they show that the angular dependence of the net excited charge presented in Fig. 3 is robust up to a critical value, delimiting the optimal intensity range in which the phenomenon can be observed.

### B. Experimental results

To experimentally investigate the role of crystal orientation in ultrafast carrier photoinjection of Ge, two ATRS datasets have been collected for the two distinct directions described in Sec. II A, while keeping all other experimental parameters fixed. The transient reflectivity trace is built by acquiring a series of XUV spectra reflected from the sample alternating the presence ( $I_{ON}$) or the absence ( $I_{\text{OFF}}$) of the IR pulse as a function of the time delay between the XUV and IR pulses, to ultimately compute the differential reflectivity as $\frac{\Delta R}{R}=\frac{I_{ON}-I_{\text{OFF}}}{I_{\text{OFF}}}$. The delay is varied in steps of 0.33 fs to resolve the oscillating features at $2\omega_{IR}$, while the energy resolution is about 20 meV close to the Fermi energy ( $E_{F}∼$ 29.5 eV). A simultaneous attosecond streaking experiment is performed using Argon atoms as a target to extract the temporal profile of the pump vector potential, $A_{IR}\left(t\right)$, and to calibrate the delay axis.^23,40^ The results are summarized in Fig. 4, where we report the average of four different datasets for each crystal orientation.

**FIG. 5.**
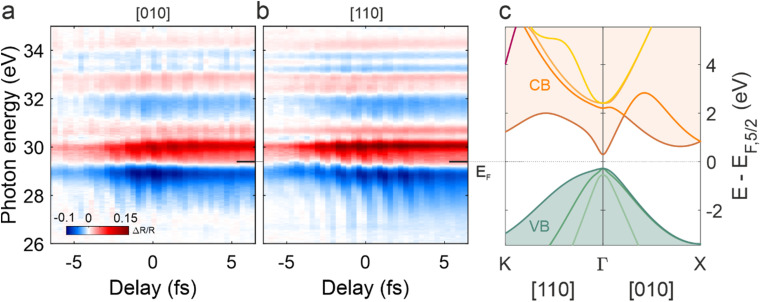
Attosecond transient reflection spectroscopy on Ge for the two crystal orientations. (a) and (b) Differential reflectivity trace for the [010] and [110] directions. (c) Band structure of Ge computed by TDDFT. Intra-band motion is along the Γ−K direction when the IR electric field is along the [110] crystallographic axis; it is along the Γ−X direction when the IR polarization is along either the [010] or the [100] direction.

The overall transient reflectance trace does not qualitatively change when the crystal is rotated from [010] [Fig. 5(a)] to [110] [Fig. 5(b)]. Indeed, for both cases, ΔR/R is characterized by areas of augmented (reduced) reflectivity, marked in red (blue), which mostly relate to those regions of the band structure [Fig. 5(c)] found to be responsible for the static optical properties of the material.^17,41,42^ The major transient features, whose physical origin is described in Ref. 17, are composed by an oscillating response at $2\omega_{IR}$ on top of a few-femtosecond signal, which either follows the IR pump envelope or monotonically builds-up during the interaction.^17^ While the latter is expected to be dominated by real carrier dynamics, the former mainly originates from the IR field transiently dressing the crystal and inducing optical Stark shift and intra-band motion.^27^ In addition to Ge,^17^ a similar pattern has been observed in GaAs,^13^ where calculations indicate intra-band motion (i.e., dynamical Franz–Keldysh effect or DFKE^43^) as the main responsible for the strong energy dispersion of the $2\omega_{IR}$ oscillations around the Fermi edge. This peculiar behavior has been also observed in dielectrics^19,20,23^ where recent results have suggested the interaction among Floquet-ladder states separated by one IR photon as the origin of the strong energy dispersion of the oscillations.^21^ Since Floquet states can form also with short pump pulses,^44,45^ we expect this phenomenon to potentially influence the sub-cycle features of the Ge transient optical response.

Despite the similarities, a closer look at the data presented in Fig. 5 reveals that the amplitude of the transient response is overall larger for the [110] case, especially in the region close to the Fermi energy, $E_{F}$, at about 29.5 eV as we will discuss in Sec. III C.

### C. Theory and experiment comparison

**FIG. 6.**
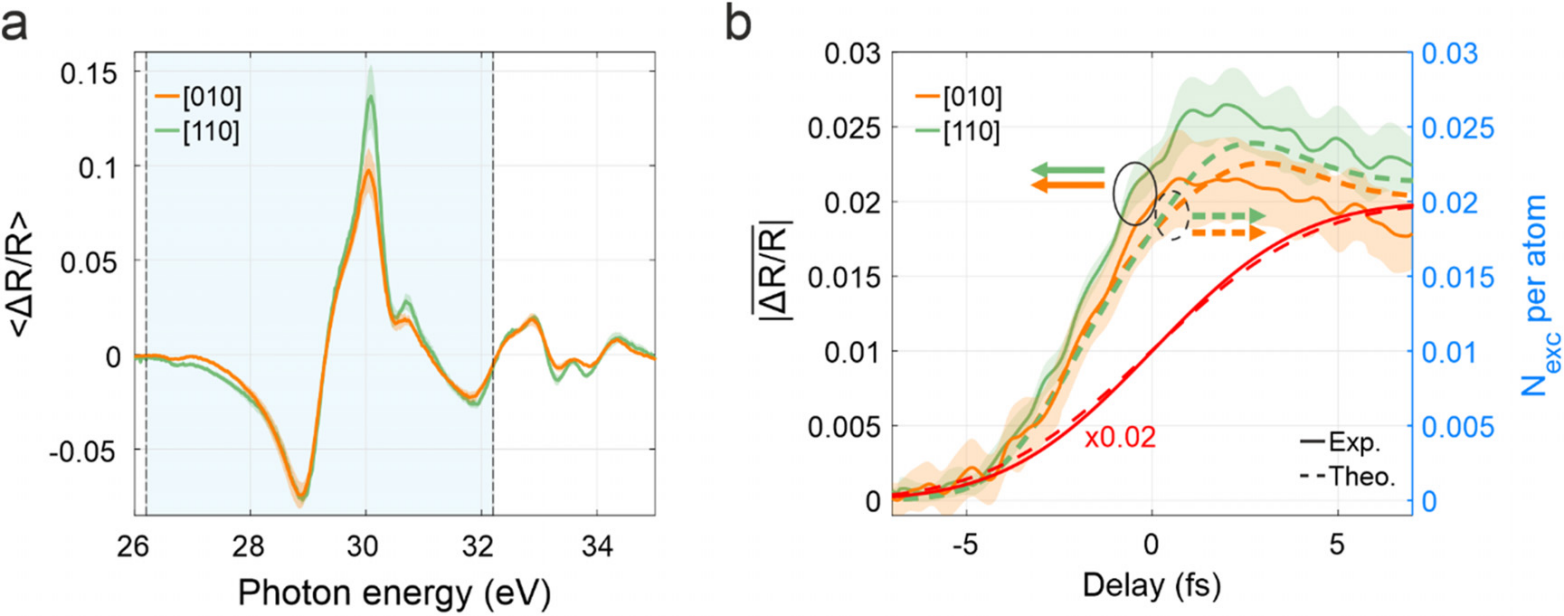
Quantitative comparison between the results of ATRS and excited population analysis. (a) Delay-averaged differential reflectivity between −7 and +7 fs for the [010], orange, and [110], green, orientations. (b) Average of the absolute value of the experimental differential reflectivity trace in the energy region marked by the shaded light-blue area in (a), $\bar{\left|\Delta R/R\right|}$, (solid curves) compared with the calculated electron population, $N_{\text{exc}}$ (dashed curves). The red curves show the normalized pump fluence as extracted from the experiment (solid) and from the calculations (dashed). In both panels, the solid curves represent the mean value while the shaded areas display the standard deviation.

The experimental results described above are thus in agreement with the qualitative robustness of the charge injection mechanism and the increased excitation efficiency for the [110] orientation observed in the TDDFT calculations. Although ΔR/R and $N_{\text{exc}}$ represent fundamentally different physical quantities, it is, therefore, conceivable to inquire whether a more quantitative comparison could be performed between the reflectivity transient features and the computed electron populations. Such a comparison is further complicated by the fact that Kramers–Kronig relations convert an energetically narrow absorption feature, easily ascribable to a defined transition, into a broad feature in the reflectivity, thus making the contribution of separate optical transitions to overlap and hindering a detailed energy-resolved study. Therefore, to proceed with our analysis, we computed the average value of $\left|\Delta R/R\right|$ in an energy range across the Fermi level, i.e., between 26.2 and 32.2 eV [light blue area in Fig. 6(a)], where the effect of the real charges injected into the CB is expected to be stronger. This energy region around $E_{F}$ contains the largest pump-induced transient signals as well as the main modification of ΔR/R due to crystal rotation [visible also in the ATRS traces integrated in delay between −7 and +7 fs and reported in Fig. 6(a)]. The resulting energy-averaged modulus of the reflectivity, $\bar{\left|\Delta R/R\right|}$, is reported in Fig. 6(b) (shaded areas represent its standard deviation). We note that the oscillating component at $2\omega_{IR}$ is drastically suppressed after the energy average due to the energy-dependent phase delay of oscillating transient signals composing the ATRS trace [Figs. 5(a) and 5(b)]. Orange colors represent the [010] orientation while green is used for the [110] case. For both orientations $\bar{\left|\Delta R/R\right|}$ does not follow the IR fluence [red curves in Fig. 6(b)], but increases, reaching a maximum roughly 2 fs after the maximum of the pump pulse envelope ( t=0 fs), with the [110] curve that stays overall above the one for [010]. This temporal evolution is strikingly similar to the one of the calculated total $N_{\text{exc}}$ presented in Fig. 3(c) and reported in Fig. 6(b) with dashed curves for a better comparison. The similar temporal trend suggests a link between the increase in the strength of the transient reflectivity features and the increased calculated excitation yield. Nevertheless, while the time integral of $N_{\text{exc}}\left(t\right)$ in the region −7 fs≤t≤7 fs increases by 3.8% in the [110] case, the time integral of $\bar{\left|\Delta R/R\right|}$ manifest a significantly larger increase for the [110] direction, which amounts to 19.5 ± 1.8% (the error was obtained by propagating the standard deviation of the mean directly extracted from ATRS data). We note that the energy dispersion of the sub-cycle oscillations increases the standard deviation associated with the experimental $\bar{\left|\Delta R/R\right|}$, causing the two experimental uncertainties to touch. However, we believe the observed difference between the two orientations is significant for two reasons: (i) the effect is clearly visible in the differential reflectivities of Fig. 5, which represent an average of single measurements taken in several days and switching back and forth between the two orientations; and (ii) the transient optical properties of Ge around the pump-probe overlap and in the region between 26 and 32 eV are dominated by the contribution coming from electron and hole excitation,^46^ which is found to be higher for the [110] case in our calculations.

While this comparison shows that ΔR/R around the Fermi edge is capable of probing subtle changes in the charge injection yield, we believe the stronger observed variation to stress the importance of the probing mechanism due to the XUV pulse. In fact, since the almost 20% increment in $\bar{\left|\Delta R/R\right|}$ cannot be simply explained in terms of a larger number of IR-excited charges in the crystal, it must be partially ascribed to a more effective probing of the excited carrier population for the [110], where the electron density along the XUV polarization axis is larger. We note that keeping the XUV polarization parallel to the [010] direction for both experiments would have caused part of the attosecond radiation to impinge onto the Ge surface with p polarization, further complicating the interpretation of the associated change in the sample reflectivity.

## IV. CONCLUSIONS

In this work, we performed TDDFT calculations and ATRS measurements to study the complex field-driven photoinjection mechanism in monocrystalline Ge, under two distinct field-lattice orientations. Our simulations show that while the different alignment between the Ge lattice and the IR field causes a different momentum distribution of the photoexcited electrons, the overall charge injection process is robust and can be investigated by grouping the involved k points with respect to the ratio between the local energy gap and the photon energy, i.e., the resonant parameter M. In agreement with previous results, for both chosen orientations we found that most of the net excited charge is injected by one or two-photon transitions which happen at larger values of momentum, moving away from the Γ point. While resonant excitation dominates on the leading edge of the pump pulse, two-photon transitions become predominant around the peak of the pump envelope, exhibiting a clear broken mirror-symmetry that is ascribed to intra-band motion. A qualitative analysis would thus suggest that the ultrafast interaction between the crystal and the field is insensitive to the orientation, but a more detailed, quantitative analysis reveals fine differences. By looking at the temporal evolution of the electron population difference between the two orientations, $\Delta N_{\text{exc}}$, we found it to differ from zero and to evolve in time in a non-monotonic manner. Before the pump pulse peak ${\Delta N}_{\text{exc}}$ is negative, indicating a more efficient excitation for the [010] case, while during the second half of the pulse, ${\Delta N}_{\text{exc}}$ becomes positive, leading to a net increased yield for the [110] direction. Surprisingly, the strongest transient contribution to the difference between the [010] and [110] orientations does not come from the dominating two-photon transitions, but from those k points located closer to Γ, with M∼1, which display a similar temporal evolution of the excited carriers. To test the picture suggested by the TDDFT results, we performed ATRS measurements for the two chosen orientations. The measurements reveal a qualitatively similar structure of transient features, characterized by fast oscillations at twice the IR frequency superimposed to a few-femtosecond signal, thus confirming the overall robustness of the underlying physical mechanisms. Moreover, an analysis of the ATRS traces around the Ge Fermi edge shows that the amplitude of the transient optical features increases for the [110] alignment and follows a temporal evolution similar to the one computed for the electron population into the CB, which is not simply proportional to the IR field fluence. Therefore, our results not only prove the capability of ATRS of detecting subtle differences in the photoinjection process of semiconductors, but also suggest that crystal orientation may be used as an additional knob to fine-tune the momentum distribution of the excited electrons and their precise timing within a 5-fs pump pulse. Further experiments based on time- and momentum-resolved techniques are needed to verify this point and further elucidate the role of crystal orientation in determining both the relative weight between different physical mechanisms and the total ultrafast charge injection efficiency. Adding a new piece to the intricated puzzle that describes real-time interaction between a strong light field and matter, our work thus moves a further step toward an aware and efficient use of ultrashort light pulses in the next-generation field-driven optoelectronics.

## Data Availability

The data that support the findings of this study are available from the corresponding authors upon reasonable request.
